# Clinical and social burden of migraine in Japanese males: a cross-sectional comparison with females without menstrual migraine

**DOI:** 10.1186/s10194-026-02405-z

**Published:** 2026-05-28

**Authors:** Tsubasa Takizawa, Keiko Ihara, Reiko Yoshikawa, Kanae Togo, Takahiro Kitano, Masahiro Iijima

**Affiliations:** 1 Department of Neurology, Keio University School of Medicine, Tokyo, Japan; 2 Internal Medicine/Hospital/Antiviral Medical Affairs, Pfizer Japan Inc., Tokyo, Japan; 3 Access & Value, Pfizer Japan Inc., Tokyo, Japan

**Keywords:** Headache, Male migraine, Migraine, Pain intensity and duration, Sex differences, Social burden

## Abstract

**Background:**

Migraine is globally more prevalent in females; however, the clinical and social burden among males remains insufficiently characterized. This study aimed to describe the clinical features and social burden of migraine in Japanese males to identify sex-specific migraine profiles.

**Methods:**

A cross-sectional, population-based web survey was conducted among Japanese adults aged ≥ 18 years diagnosed with migraine according to modified criteria from the International Classification of Headache Disorders, Third Edition (ICHD-3). Participants were grouped by sex; females diagnosed with menstrual migraine (MM) were excluded to minimize hormonal confounding. Key outcomes included pain intensity (Visual Analog Scale [VAS]), pain duration, and social burden assessed using validated instruments: Migraine Interictal Burden Scale (MIBS-4), Migraine Disability Assessment (MIDAS), Migraine-Specific Quality of Life Questionnaire (MSQ), and Work Productivity and Activity Impairment for Migraine (WPAI-M) scores.

**Results:**

Of 266,392 individuals screened, 18,750 completed the survey; 4,161 males and 9,997 females (excluding those with MM) were included in the analysis. Males were older (mean± standard deviation age: 43.9 ± 12.4 vs. 40.9 ± 12.2 years) and reported more lifestyle-related triggers (e.g., alcohol, irregular eating, and overexercise). Males, compared to females, experienced greater pain intensity (VAS: 57.1 ± 16.7 vs. 54.9 ± 19.6; *p* < 0.001) but shorter pain duration (6.3 ± 9.4 vs. 7.9 ± 12.4 h; *p* < 0.001). MIDAS scores were comparable (6.3 ± 10.8 vs. 6.0 ± 9.6; *p* = 0.283), while MIBS-4 scores were higher in males (4.0 ± 3.5 vs. 3.3 ± 3.3; *p* < 0.001). MSQ scores were lower in males for domains of interference (83.5 ± 20.0 vs. 85.4 ± 18.3; *p* = 0.001) and emotional function (80.2 ± 20.9 vs. 81.8 ± 20.3; *p* = 0.007). Work-related impairment was more pronounced in males than in females, with higher absenteeism (4.7 ± 14.1 vs. 3.4 ± 12.7; *p =* 0.001), presenteeism (31.5 ± 22.9 vs. 29.5 ± 23.1; *p* = 0.007), and overall work impairment (33.6 ± 24.6 vs. 30.9 ± 24.3; *p =* 0.001). Preventive medication use was higher in males (oral: 40.8% vs. 34.3%; injectables: 3.3% vs. 1.7%).

**Conclusion:**

Males with migraine exhibited a greater burden in terms of pain intensity and functional impairment relative to females without MM. These findings support the development of sex-specific and individualized management strategies that incorporate both pharmacological and lifestyle interventions to reduce disease burden and enhance patient-centered care.

**Trial registration:**

Not applicable.

**Supplementary Information:**

The online version contains supplementary material available at 10.1186/s10194-026-02405-z.

## Introduction

Migraine is a prevalent and disabling neurological disorder, affecting over one billion individuals worldwide and contributing significantly to global disability-adjusted life years [1]. Although migraine is more common in females—with approximately 18% affected compared with 6% of males [2]—recent data suggest that the rate of increase in migraine prevalence among males has outpaced that of females in recent decades [1]. In Japan, migraine is three to four times more prevalent in females than in males [3].

Most evidence reflects the sex disparity in migraine [4–8]. Additionally, the influence of sex hormones, particularly estrogen fluctuations, on migraine pathophysiology in females has been well documented. Hormonal factors, including fluctuations in estrogen levels, have been shown to influence migraine characteristics in females, particularly in the context of menstrual migraine (MM) [9–11]. Consequently, MM is defined in the appendix of the International Classification of Headache Disorders, Third Edition (ICHD-3) [12], and is characterized by more severe headache intensity and longer duration [2].

Despite the predominance of female-focused research, males also experience a substantial disease burden [13–15]. A recent cross-sectional survey in Poland showed significant sex differences in migraine clinical characteristics and medical care [13]. It suggested that considering sex-related determinants of migraine may aid in individualized medical strategies that could contribute to improving the quality of care in migraine [13]. In contrast, the clinical and social burden of migraine in males has been relatively underexplored, despite evidence of substantial impairment and distinct clinical characteristics, limiting a comprehensive understanding of sex-related differences beyond hormonally driven mechanisms.

Moreover, males are more likely to experience high-frequency migraine (> 10 monthly headache days [MHD]) and have a later age of onset compared with females [15, 16]. A large claims database study in Japan revealed that males account for 35% of migraine patients in the country [14]. However, most existing studies in Japan have centered on female populations, particularly those affected by hormonal influences, leaving a gap in understanding the clinical and social burden of migraine in males.

This study aims to characterize the clinical features and evaluate the social burden of migraine in Japanese males, using a comparative approach with females unaffected by MM, to address the existing knowledge gap regarding sex-specific differences in the clinical and social burden of migraine in real-world settings.

Our previous study demonstrated comparison between MM and non-MM [17]. Therefore, to omit the impact of menstruation-related migraine in the analysis, and to identify sex-specific differences in the migraine burden, we aimed to conduct this study by excluding females with MM.

## Methods

### Study design

This was a cross-sectional, population-based web survey conducted among Japanese patients with migraine [17]. Participants were randomly selected from a registry (Macromill Carenet Inc.) comprising over 1.3 million individuals (aged 20–59 years: 83%; males: 34%). Eligible participants were identified based on a screening assessment questionnaire and invited to complete the survey through the registry’s online platform. During sampling, the proportions of sex and age groups were adjusted to ensure that the study population demographics were representative of Japanese patients with migraine. Data were collected between November 11 and 29, 2024. This study is reported in accordance with the STROBE guidelines for observational studies.

### Study population

This study included male and female participants aged ≥ 18 years at the time of the survey who had a migraine diagnosis (with or without aura), defined according to the modified ICHD-3 criteria, and provided electronic informed consent for participation. Participants were excluded if they were receiving sex hormone therapy (e.g., for conditions such as endometriosis, dysmenorrhea, or prostate cancer); were pregnant; or had ≥ 15 migraine days/month, psychiatric or neurological disorders (including major depressive disorder or epilepsy), secondary headaches, cluster headaches, or any medical conditions or concomitant use of medications that could potentially influence the pathophysiology or pain characteristics of migraine (e.g., cancer-related pain, fibromyalgia, chronic pelvic pain, or complex regional pain syndrome).

Eligible participants were categorized into two groups: male and female. The female group excluded patients with MM−defined as those experiencing migraine attacks during the perimenstrual period (days −2 to +3 of the onset of menstruation) in at least two out of three menstrual cycles [12, 17]. Females without MM were selected to minimize the confounding effects of hormonal influences associated with MM, enabling a more accurate comparison with the male group, who experience migraine independent of such hormonal influences. Thus, data from male participants (male group) and female participants with migraine unrelated to menstruation (female group) were analyzed in this study. The comparison between MM and non-MM in female participants has been reported previously [17]. This analysis includes females without MM, some of whom were reported previously. Specifically, 5,174 of the 9,997 females without MM (51.8%) overlap with the non-MM cohort described in the prior publication.

### Study outcomes and assessment

Baseline demographics included age, sex, body mass index (BMI), employment status, personal and family income, smoking habits, and alcohol consumption. Clinical characteristics comprised patient medical history, family history of migraine, and associated symptoms. Additional features included age at migraine onset, classification of migraine type (with or without aura), monthly migraine days (MMD), MHD, duration of migraine episodes, and identified migraine triggers. Outcomes included pain intensity, pain duration, and social burden. Pain intensity was assessed using the Visual Analog Scale (VAS). The social burden of migraine was assessed using the Migraine Interictal Burden Scale (MIBS-4) [18], Migraine Disability Assessment (MIDAS) [19, 20], Migraine-Specific Quality of Life Questionnaire (MSQ) [21], and Work Productivity and Activity Impairment for Migraine (WPAI-M) scores [22]. Medication status included use of prescribed acute and preventive treatments, number of days of prescription use for acute treatment, over-the-counter (OTC) medication use, and frequency of OTC medication use.

### Statistical analysis

Data from all eligible participants who completed the survey were analyzed. Patient characteristics and medication use were summarized using descriptive statistics. Patient characteristics were compared between the male and female groups using t-tests for continuous variables and Fisher’s exact test for categorical variables at a significance level of 0.05. Outcomes including pain intensity (VAS score); migraine attack duration; MIDAS, MIBS-4, and MSQ scores; and WPAI-M scores were summarized descriptively. The 95% confidence intervals for means or percentages were also calculated.

Comparisons between the groups were made using multivariate regression models, adjusting for potential confounding factors using linear regression models with inverse probability of treatment weighting (IPTW) [23]. The propensity scores for IPTW were estimated using a logistic regression model for the male and female groups, with potential confounders including age, BMI, smoking habits, alcohol intake, age at migraine onset, treatment status, employment status, and MMD; for IPTW, stabilized weights were used. These covariates were selected based on a previous study [24], along with additional variables considered relevant to the severity of migraine from a clinical perspective, to adjust for potential confounders.

## Results

### Patient selection and group creation

**Fig. 1:**
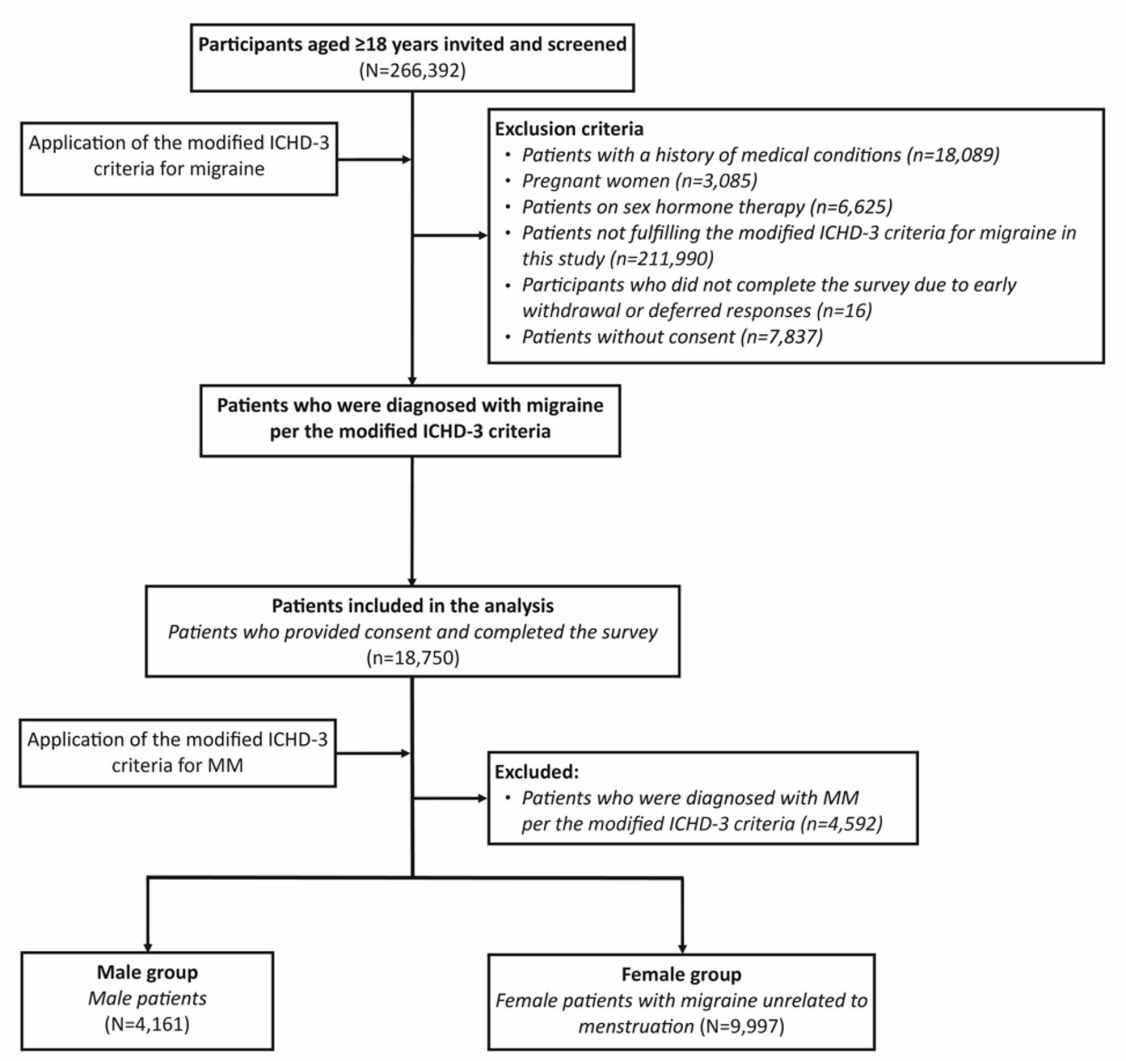
Patient disposition and group creation. ICHD-3, International Classification of Headache Disorders, Third Edition; MM, menstrual migraine. Note: The study excluded patients with MM (*n* = 4,592), defined as those experiencing migraine attacks during the perimenstrual period (days −2 to +3 of the onsets of menstruation) in at least two out of three menstrual cycles

Patient selection and the study groups are presented in Fig. 1. A total of 266,392 patients were invited and screened. Of these, patients were excluded if they had a history of certain medical conditions or if they had psychiatric disorders or epilepsy (*n* = 18,089), were pregnant (*n* = 3,085), were receiving sex hormone therapy (*n* = 6,625), were not diagnosed with migraine (*n* = 211,990), did not complete the survey due to early withdrawal or deferred responses (*n* = 16), or did not provide consent (*n* = 7,837). Overall, 18,750 patients provided consent and completed the survey. Of these, this study included the male group (*n* = 4,161), comprising male patients with an ICHD-3 diagnosis of migraine, and the female group (*n* = 9,997), comprising female patients with a migraine diagnosis unrelated to menstruation.

### Patient characteristics

Patient characteristics of the female and male groups are summarized in Table 1. The mean± standard deviation (SD) age was significantly higher in the male group than in the female group (43.9 ± 12.4 years vs. 40.9 ± 12.2 years; *p* < 0.001). The age at migraine onset was also higher in the male group than in the female group (23.2 ± 11.9 years vs. 20.3 ± 9.5 years; *p* < 0.001). The mean MHD and MMD were 4.5 ± 4.0 and 2.9 ± 2.4 in the male group and 4.8 ± 4.2 and 2.8 ± 2.4 in the female group, respectively. A history of hypertension (42.6% vs. 11.0%), sleep disorders (24.4% vs. 9.9%), anxiety (15.5% vs. 9.5%), and diabetes (13.6% vs. 3.7%) was more common in the male group than in the female group (all *p* < 0.001). A significantly higher proportion of patients experienced aura in the male group than in the female group (29.8% vs. 24.7%; *p* < 0.001). The proportion of phonophobia (47.2% vs. 52.6%), photophobia (45.1% vs. 52.0%), and osmophobia (15.7% vs. 22.5%) was significantly lower (all *p* < 0.001) in the male than in the female group. The proportion of patients who visited a doctor or hospital for a formal migraine diagnosis was significantly higher in the male group than in the female group (34.7% vs. 30.2%; *p* < 0.001). Additionally, significantly more males reported smoking and alcohol consumption compared with females (current smokers: 30.2% vs. 10.7%; daily alcohol consumption: 21.9% vs. 8.7%; both *p* < 0.001) (Supplementary Table 1).

**Table 1:** Patient demographics and clinical characteristics

Characteristics	Male group (*N* = 4,161)	Female group (*N* = 9,997)	*p*-value*
*Age (years)*			
Mean ± SD	43.9 ± 12.4	40.9 ± 12.2	< 0.001
*Age at migraine onset (years)*			
Mean ± SD	23.2 ± 11.9	20.3 ± 9.5	< 0.001
*BMI (kg/m* ^*2*^ *)*			
Mean ± SD	23.1 ± 3.8	21.6 ± 4.2	< 0.001
< 25	3,138 (75.4)	8,428 (84.3)	< 0.001
≥ 25 to < 30	823 (19.8)	1,156 (11.6)	
≥ 30	200 (4.8)	413 (4.1)	
*MHD*			
Mean ± SD	4.5 ± 4.0	4.8 ± 4.2	< 0.001
*MMD*			
Mean ± SD	2.9 ± 2.4	2.8 ± 2.4	0.336
*Medical history*			
n	1,836	5,306	
Sleep disorder	448 (24.4)	526 (9.9)	< 0.001
Anxiety	284 (15.5)	504 (9.5)	< 0.001
Gastric ulcer/gastrointestinal bleeding	72 (3.9)	44 (0.8)	< 0.001
Diabetes	250 (13.6)	195 (3.7)	< 0.001
Hypertension	782 (42.6)	582 (11.0)	< 0.001
Uterine fibroids	NA	783 (14.8)	NA
Endometriosis	NA	388 (7.3)	NA
Adenomyosis uteri	NA	129 (2.4)	NA
Gynecological tumor	NA	142 (2.7)	NA
Dysmenorrhea	NA	399 (7.5)	NA
PMS	NA	1,614 (30.4)	NA
*Aura*	1,238 (29.8)	2,467 (24.7)	< 0.001
*Associated symptoms*			
Nausea	2,213 (53.2)	5,880 (58.8)	< 0.001
Vomiting	481 (11.6)	1,721 (17.2)	< 0.001
Phonophobia	1,964 (47.2)	5,261 (52.6)	< 0.001
Photophobia	1,878 (45.1)	5,194 (52.0)	< 0.001
Osmophobia	654 (15.7)	2,251 (22.5)	< 0.001
*Triggers for migraine*			
Stress	1,988 (47.8)	4,700 (47.0)	0.408
Relief from stress	424 (10.2)	1,046 (10.5)	0.627
Menstruation	NA	2,950 (29.5)	NA
Emotional ups and downs (overexcitement or mood swings)	503 (12.1)	1,380 (13.8)	0.006
Fatigue and malaise	1,995 (47.9)	5,189 (51.9)	< 0.001
Seasonal transitions	1,343 (32.3)	3,507 (35.1)	0.001
Weather change	1,633 (39.2)	5,612 (56.1)	< 0.001
Poor posture	827 (19.9)	2,192 (21.9)	0.007
Overexercise	283 (6.8)	437 (4.4)	< 0.001
Irregular eating habits	295 (7.1)	378 (3.8)	< 0.001
Alcohol consumption	643 (15.5)	978 (9.8)	< 0.001
Caffeine consumption	207 (5.0)	420 (4.2)	0.042
Consumption of specific foods	68 (1.6)	149 (1.5)	0.526
Exposure to light	566 (13.6)	1,583 (15.8)	< 0.001
Noise	261 (6.3)	875 (8.8)	< 0.001
Medication	59 (1.4)	97 (1.0)	0.020
Irregular sleep	1,497 (36.0)	3,545 (35.5)	0.559
Certain smells (e.g., gasoline or perfume)	322 (7.7)	1,052 (10.5)	< 0.001
Not sure/no trigger	569 (13.7)	1,342 (13.4)	0.691

*t-tests for continuous variables and Fisher’s exact test for categorical variables were performed at a significance level of 0.05

Data are presented as n (%), unless otherwise specified

BMI, body mass index; MHD, monthly headache days; MMD, monthly migraine days; NA, not applicable; PMS, premenstrual syndrome; SD, standard deviation

### Pain intensity and duration

The VAS score (mean ± SD, after IPTW adjustment) for migraine pain intensity was significantly higher in the male group than in the female group (57.1 ± 16.7 vs. 54.9 ± 19.6; *p* < 0.001). The duration of migraine attacks (mean ± SD, after IPTW adjustment) was significantly shorter in the male group than in the female group (6.3 ± 9.4 vs. 7.9 ± 12.4; *p* < 0.001) (Table 2). After IPTW, standard mean differences (SMD) for all covariates between the compared groups were < 0.1 except for two variables (age and age of migraine onset). For age and age of migraine onset, the SMDs were 0.24 and 0.14, respectively (Supplementary Table 2).

**Table 2:** Comparison of pain intensity and pain duration between the male and female groups

Outcomes		Before IPTW adjustment		After IPTW adjustment		Difference between the groups with IPTW	
		Male group (*N* = 4,161)	Female group (*N* = 8,527)	Male group (ESS = 1,688)	Female group (ESS = 8,826)	Mean difference (95% CI)	*p*-value
Pain intensity (VAS score)	Mean ± SD	57.7 ± 16.6	54.6 ± 19.5	57.1 ± 16.7	54.9 ± 19.6	2.2 (1.3, 3.1)	< 0.001
Pain duration (hours)	Mean ± SD	6.1 ± 9.1	7.8 ± 12.3	6.3 ± 9.4	7.9 ± 12.4	−1.5 (−2.1, −1.0)	< 0.001

The mean difference (95% CI) and *p*-values are presented for the outcomes of the comparison between the male and female groups after adjustment using IPTW

CI, confidence interval; ESS, effective sample size; IPTW, inverse probability of treatment weighting; SD, standard deviation; VAS, Visual Analog Scale

### Triggers

Patients in the male group vs. female group reported weather change (39.2% vs. 56.1%), fatigue and malaise (47.9% vs. 51.9%), exposure to light (13.6% vs. 15.8%), certain smells (7.7% vs. 10.5%), and noise (6.3% vs. 8.8%) as primary triggers for migraine attacks. Patients in the male group vs. the female group more frequently reported alcohol consumption (15.5% vs. 9.8%), irregular eating habits (7.1% vs. 3.8%), and overexercise (6.8% vs. 4.4%) as major triggers for migraine attacks. While stress and irregular sleep were commonly reported triggers in both groups (after fatigue and malaise), the proportions were similar between groups (Table 1).

### Medication status

The medication status of patients in the male and female groups is presented in Table 3. Overall, 33.7% of male patients and 31.7% of female patients were prescribed acute medications. A comparable proportion of patients among those prescribed acute medications in both groups were prescribed specific classes of acute medications as follows: non-steroidal anti-inflammatory drugs (NSAIDs; male group: 43.3%; female group: 46.6%), acetaminophen (37.5%; 40.6%), and triptans (22.0%; 24.5%). A slightly lower proportion of patients in the male group used OTC medications compared with the female group (76.4% vs. 78.1%). Preventive medication use was higher among patients in the male group than in the female group (oral: 40.8% vs. 34.3%; injectables: 3.3% vs. 1.7%).

**Table 3:** Medication status

Medication status	Male group (*N* = 4,161)	Female group (*N* = 9,997)
*Prescription of acute medication*		
Yes	1,403 (33.7)	3,165 (31.7)
No	643 (15.5)	1,134 (11.3)
I have not been to the hospital	2,115 (50.8)	5,698 (57.0)
*Description of the prescribed acute medication*		
n	1,403	3,165
Triptan	308 (22.0)	777 (24.5)
Lasmiditan	59 (4.2)	52 (1.6)
Acetaminophen	526 (37.5)	1,286 (40.6)
NSAIDs	607 (43.3)	1,475 (46.6)
Prescribed but not sure of its classification	252 (18.0)	448 (14.2)
*Prescription of acute medication (days per month)*		
n	1,403	3,165
Mean ± SD	3.4 ± 3.2	3.3 ± 3.4
*OTC drug use (days per month)*		
n (%)	3,177 (76.4)	7,806 (78.1)
Mean ± SD	3.0 ± 3.0	3.1 ± 3.2
*Prescription of oral preventive medication*		
n	2,046	4,299
Yes	835 (40.8)	1,473 (34.3)
*Prescription of injectable preventive medication*		
n	2,046	4,299
Yes	68 (3.3)	72 (1.7)

Data are presented as n (%), unless otherwise specified

NSAID, non-steroidal anti-inflammatory drug; OTC, over-the-counter; SD, standard deviation

### Social burden

The MIBS-4 score (mean ± SD, after IPTW adjustment) was significantly higher in the male group than in the female group (4.0 ± 3.5 vs. 3.3 ± 3.3; *p* < 0.001). The MIDAS score was similar between the male and female groups (6.3 ± 10.8 vs. 6.0 ± 9.6; *p* = 0.283). The MSQ scores for interference and emotion were significantly lower in the male group than in the female group (interference: 83.5 ± 20.0 vs. 85.4 ± 18.3; *p* = 0.001; emotional function: 80.2 ± 20.9 vs. 81.8 ± 20.3; *p* = 0.007). However, the MSQ score (mean ± SD, after IPTW adjustment) for restriction was similar between groups (76.3 ± 19.3 vs. 77.3 ± 17.9; *p* = 0.090). The WPAI-M scores showed that the male group experienced significantly higher overall work impairment (33.6 ± 24.6 vs. 30.9 ± 24.3; *p* = 0.001), absenteeism (4.7 ± 14.1 vs. 3.4 ± 12.7; *p* = 0.001), and presenteeism (31.5 ± 22.9 vs. 29.5 ± 23.1; *p* = 0.007) compared with the female group. However, impairment in daily activities was similar between groups (31.3 ± 22.4 vs. 31.1 ± 23.3; *p* = 0.675) (Table 4).

**Table 4:** Social burden of migraine in the male and female groups

Outcomes	Before IPTW adjustment		After IPTW adjustment		Difference between the groups with IPTW	
	Male group (*N* = 4,161)	Female group (*N* = 9,997)	Male group (ESS = 1,688)	Female group (ESS = 8,826)	Mean difference (95% CI)	*p*-value
*Migraine burden level*						
MIBS-4 score	4.0 ± 3.4	3.3 ± 3.2	4.0 ± 3.5	3.3 ± 3.3	0.7 (0.5, 0.9)	< 0.001
*Labor productivity (WPAI-M score)*						
Percentage of work hours missed (absenteeism)*	5.0 ± 14.3	3.2 ± 12.4	4.7 ± 14.1	3.4 ± 12.7	1.3 (0.5, 2.0)	0.001
Percentage of work impairment (presenteeism)^†^	32.4 ± 22.4	29.2 ± 23.0	31.5 ± 22.9	29.5 ± 23.1	1.9 (0.5, 3.4)	0.007
Percentage of overall work impairment^†^	34.6 ± 24.2	30.5 ± 24.1	33.6 ± 24.6	30.9 ± 24.3	2.7 (1.2, 4.2)	0.001
Percentage of daily activity impairment	31.5 ± 22.1	31.1 ± 23.3	31.3 ± 22.4	31.1 ± 23.3	0.3 (−0.9, 1.4)	0.675
*Migraine-related disabilities (MIDAS score)*						
MIDAS score	5.9 ± 9.7	5.9 ± 9.5	6.3 ± 10.8	6.0 ± 9.6	0.3 (−0.3, 0.9)	0.283
*Migraine-specific quality of life (MSQ score)*						
Limit/restriction	77.3 ± 17.8	77.3 ± 17.8	76.3 ± 19.3	77.3 ± 17.9	−0.9 (−2.0, 0.1)	0.090
Interference	84.1 ± 18.6	85.4 ± 18.3	83.5 ± 20.0	85.4 ± 18.3	−1.9 (−3.0, −0.8)	0.001
Emotional function	80.8 ± 19.7	81.9 ± 20.3	80.2 ± 20.9	81.8 ± 20.3	−1.6 (−2.7, −0.4)	0.007

*n = 3,439 and 6,566 for the male group and female group, respectively

^†^*n* = 3,415 and 6,518 for the male group and female group, respectively

All values are presented as mean ± SD. The mean difference (95% CI) and *p*-values are presented for the outcomes of the comparison between the male and female groups after adjustment using IPTW

CI, confidence interval; ESS, effective sample size; IPTW, inverse probability of treatment weighting; MIBS-4, Migraine Interictal Burden Scale; MIDAS, Migraine Disability Assessment; MSQ, Migraine-Specific Quality of Life Questionnaire; SD, standard deviation; WPAI-M, Work Productivity and Activity Impairment for Migraine

## Discussion

This cross-sectional study presents a comprehensive analysis of the clinical characteristics, medication patterns, and social burden associated with migraine in male patients in Japan, compared with female patients without MM. While previous research has predominantly focused on females—often irrespective of the presence of MM, which is influenced by estrogen fluctuations and may affect pain severity, duration, and associated social burden—this study highlights important distinctions in the male population.

Notably, males demonstrated a higher prevalence of aura and reported fewer associated symptoms and higher comorbidities, such as hypertension, sleep disorders, and anxiety, as well as a greater number of lifestyle-related triggers (overexercise, irregular eating, and alcohol) relative to females without MM. Furthermore, male patients indicated higher pain intensity on the VAS and experienced a more pronounced migraine-related burden, as evidenced by MIBS-4, MSQ, and WPAI-M scores. These findings may be partially attributable to the increased hospital visits (males, 34.7% vs. females, 30.2%; *p* < 0.001; Supplementary Table 1) and utilization of injectable CGRP-associated monoclonal antibodies (injectable preventive medication prescription: males, 3.3% vs. females; 1.7%; Table 3) among male patients. Such trends could reflect more severe disease, higher employment rates (housewife/househusband: males, 0.6% vs. females, 21.5% and part-time job, 5.6% vs. 25.9%; *p* < 0.001; Supplementary Table 1), and greater income levels (personal income < 2 million yen (< 12,922 USD): males, 13.5% vs. females, 47.1%; *p* < 0.001; Supplementary Table 1) among males in Japan.

Aura was more prevalent in the male group, whereas symptoms such as photophobia and phonophobia occurred less frequently. These observations align with recent data from the Leiden Headache Clinic, which identified a higher prevalence of migraine with aura in males than in females, for both perimenstrual and non-perimenstrual [8]. Given the higher incidence of aura among males, clinicians should adopt targeted and thorough inquiry to prevent underdiagnosis or misclassification.

This study also found a higher prevalence of hypertension, diabetes, anxiety, and sleep disorders among male patients. While Tietjen et al. previously characterized males as experiencing higher comorbidities such as hypertension, hyperlipidemia, diabetes mellitus, and hypothyroidism and females as having more somatic and psychiatric symptoms [15], our results suggest a more intricate pattern in males, especially regarding sleep disturbances and anxiety. Among Japanese patients with migraine, male patients may be more likely to have comorbidities, which should be taken into consideration when planning treatment.

The onset age of migraine was slightly greater in males than in females, corroborating an earlier study [15]. In females, hormonal factors—most notably estrogen—are implicated in earlier migraine onset [10], with longitudinal data linking earlier menarche to heightened migraine risk [9, 10]. Trigger profiles, in our study, also varied by sex: females more frequently reported environmental triggers such as weather change, light, and noise, while males identified lifestyle-related factors, including alcohol use, irregular eating habits, and overexertion. It was hypothesized that sex hormonal differences may contribute to a different pattern of fluctuations in neuronal brain excitability and the internal threshold, and therefore contribute to an increased potential of external trigger factors to provoke migraine in females than in males [25]. Although the exact pathophysiological mechanism is unknown, the hormonal influence on nociceptive processing could be of great importance [7, 25]. In our study, both groups commonly cited stress and irregular sleep as triggers. Given the predominance of lifestyle-driven triggers in males, management approaches should extend beyond pharmacological interventions to encompass behavioral modifications and lifestyle counseling. Reporting bias must also be considered, as males may be more likely to identify alcohol as a trigger due to higher consumption, whereas females might underreport it.

MHD and MMD differed between males and females without MM, with males exhibiting comparable MHD and MMD. Findings from the American Migraine Prevalence and Prevention (AMPP) study similarly showed a higher proportion of males experiencing frequent migraine headaches [6]. In our study, males reported higher pain intensity on the VAS, in contrast to earlier reports indicating lower pain intensity in males [7, 10]. The difference could be influenced by the exclusion of female patients with MM, who are generally considered to have higher VAS scores, in our study [13, 17]. Although MIDAS scores were similar across groups, the higher VAS scores suggest an increased perceived pain severity among males, emphasizing the importance of individualized pain assessment. Male patients also experienced a greater overall migraine burden, including more substantial work-related impairment and decreased quality of life, as indicated by higher MIBS-4 and WPAI-M scores and lower MSQ scores. These results expand upon existing data by identifying a distinct burden profile in male migraine patients, particularly concerning pain intensity and work-related impairment.

In terms of healthcare utilization and medication patterns, male patients were more likely to seek formal medical care and receive prescription medications, including preventive therapies. Higher treatment utilization among males may reflect both migraine severity and greater access to healthcare related to socioeconomic factors and should therefore be interpreted with caution. This contrasts with earlier reports that females are more inclined to seek hospital visits and prescriptions [26]. The similar use of prescription medications (NSAIDs, acetaminophen, and triptans) and greater use of preventive medications, including injectables in males may signal better access to formal care, possibly due to higher levels of employment and income. It further suggests more frequent clinical engagement and efforts to improve quality of life among male patients.

Several limitations should be acknowledged. The study’s reliance on self-reported, cross-sectional online survey data may introduce recall and reporting biases. The absence of clinical validation for symptoms and treatment adherence limits interpretability, and recruitment methods may have introduced selection bias, potentially affecting the representativeness of the sample. Certain exclusion criteria should be considered. Patients with comorbidities including psychiatric or neurological disorders (including major depressive disorder or epilepsy), underlying medical conditions or concomitant medications (e.g., cancer-related pain, fibromyalgia, chronic pelvic pain, or complex regional pain syndrome) were excluded as it could potentially influence the pathophysiology, migraine severity, or pain characteristics of migraine. Patients with ≥ 15 MMD were also excluded due to the higher risk of medication-overuse headache. Therefore, the study population represents a restricted subgroup of patients with migraine, and the generalizability of the findings may be limited. Although IPTW was applied to adjust for confounding variables, two variables (age and age of migraine onset) were not adequately balanced, which may have influenced the results of our analysis. Age and age at migraine onset may have had nonlinear effects or interactions with other variables and addressing confounding while accounting for these complexities remains an important topic for future research. In addition, residual bias arising from unmeasured factors and self-reported diagnoses may still impact the generalizability of the findings. Future research using longitudinal designs, clinical evaluations, and randomized sampling is needed to validate and build upon these results.

## Conclusion

This study offers novel insights into the clinical presentation, treatment patterns, and social burden of migraine in male patients in Japan, compared with female patients without MM. Male patients with migraine exhibited a trend toward a higher burden in terms of pain intensity and functional impairment relative to females without MM. These findings support the development of sex-specific, individualized management strategies in migraine that incorporate both pharmacological and lifestyle interventions to reduce disease burden and enhance patient-centered care in Japan.

## Electronic supplementary material

Below is the link to the electronic supplementary material.

Supplementary Material 1

## Data Availability

All data supporting the findings of this study are available within the paper and its supplementary information.

## Abbreviations

ICHD-3: International Classification of Headache Disorders, Third Edition
MM: Menstrual migraine
VAS: Visual Analog Scale
MIBS-4: Migraine Interictal Burden Scale
MIDAS: Migraine Disability Assessment
MSQ: Migraine-Specific Quality of Life Questionnaire
WPAI-M: Work Productivity and Activity Impairment for Migraine
CGRP: Calcitonin gene-related peptide
MHD: Monthly headache days
MMD: Monthly migraine days
OTC: Over-the-counter
IPTW: Inverse probability of treatment weighting
MINS-REC-240229: MINS Research Ethics Committee
SMD: Standard mean differences
AMPP: American Migraine Prevalence and Prevention
