# Supplementary material for: Clinical and social burden of migraine in Japanese males: a cross-sectional comparison with females without menstrual migraine
Source: J Headache Pain. 2026 May 28;27(1):191. doi: 10.1186/s10194-026-02405-z (PMC13403815; doi:10.1186/s10194-026-02405-z)
Supplement: Supplementary file 1 — Supplementary Material 1 [file 10194_2026_2405_MOESM1_ESM.docx]

**SUPPLEMENTARY MATERIAL**

**Supplementary Table 1 Patient demographics and clinical characteristics**

| **Characteristics** | **Male group**  **(N=4,161)** | **Female group**  **(N=9,997)** | ***p*-value*** |
| --- | --- | --- | --- |
| *Hospital visits for diagnosis of headache* | | | |
| Migraine | 1,445 (34.7) | 3,017 (30.2) | <0.001 |
| Tension-type headache | 386 (9.3) | 973 (9.7) | 0.401 |
| No formal diagnosis/never been to the hospital | 2,548 (61.2) | 6,631 (66.3) | <0.001 |
| *Personal income* | | | |
| Less than 2 million yen | 563 (13.5) | 4,712 (47.1) | <0.001 |
| Less than 2-4 million yen | 926 (22.3) | 2,498 (25.0) |  |
| Less than 4-6 million yen | 1,174 (28.2) | 902 (9.0) |  |
| Less than 6-8 million yen | 613 (14.7) | 201 (2.0) |  |
| Less than 8-10 million yen | 324 (7.8) | 65 (0.7) |  |
| Less than 10-12 million yen | 103 (2.5) | 21 (0.2) |  |
| Less than 12-15 million yen | 59 (1.4) | 8 (0.1) |  |
| Less than 15-20 million yen | 21 (0.5) | 5 (0.1) |  |
| More than 20 million yen | 22 (0.5) | 2 (0.0) |  |
| I don’t know | 72 (1.7) | 473 (4.7) |  |
| I don’t want to answer | 284 (6.8) | 1,110 (11.1) |  |
| *Family income* | | | |
| Less than 2 million yen | 220 (5.3) | 538 (5.4) | <0.001 |
| Less than 2-4 million yen | 654 (15.7) | 1,619 (16.2) |  |
| Less than 4-6 million yen | 945 (22.7) | 1,823 (18.2) |  |
| Less than 6-8 million yen | 706 (17.0) | 1,362 (13.6) |  |
| Less than 8-10 million yen | 453 (10.9) | 756 (7.6) |  |
| Less than 10-12 million yen | 242 (5.8) | 322 (3.2) |  |
| Less than 12-15 million yen | 133 (3.2) | 166 (1.7) |  |
| Less than 15-20 million yen | 87 (2.1) | 81 (0.8) |  |
| More than 20 million yen | 29 (0.7) | 45 (0.5) |  |
| I don’t know | 326 (7.8) | 1,493 (14.9) |  |
| Missing value | 366 (8.8) | 1,792 (17.9) |  |
| *Employment status* | | | |
| Public servant | 290 (7.0) | 270 (2.7) | <0.001 |
| Manager or executive | 63 (1.5) | 26 (0.3) |  |
| Company employee (clerical) | 686 (16.5) | 1,690 (16.9) |  |
| Company employee (engineering) | 998 (24.0) | 543 (5.4) |  |
| Company employee (other) | 982 (23.6) | 1,312 (13.1) |  |
| Independent business | 244 (5.9) | 182 (1.8) |  |
| Self-employed professional (eg, actor or artist) | 99 (2.4) | 111 (1.1) |  |
| Housewife (or househusband) | 26 (0.6) | 2,153 (21.5) |  |
| Part-time job | 235 (5.6) | 2,594 (25.9) |  |
| Heian period of a student of government administration | 172 (4.1) | 542 (5.4) |  |
| Other | 97 (2.3) | 225 (2.3) |  |
| Unemployed | 269 (6.5) | 349 (3.5) |  |
| *Family structure* | | | |
| Never married, no children | 1,619 (38.9) | 3,105 (31.1) | <0.001 |
| Never married, with children | 136 (3.3) | 748 (7.5) |  |
| Married, no children | 452 (10.9) | 1,152 (11.5) |  |
| Married, with children | 1,954 (47.0) | 4,992 (49.9) |  |
| *Family history of migraine* | | | |
| Father/mother | 781 (18.8) | 1,539 (15.4) | <0.001 |
| Grandfather/grandmother | 70 (1.7) | 141 (1.4) | 0.224 |
| Brother/sister | 224 (5.4) | 580 (5.8) | 0.327 |
| Child | 90 (2.2) | 377 (3.8) | <0.001 |
| Other relatives | 45 (1.1) | 92 (0.9) | 0.372 |
| None | 1,359 (32.7) | 3,453 (34.5) | 0.031 |
| Not sure | 1,758 (42.2) | 4,309 (43.1) | 0.350 |
| *Smoking status* | | | |
| Current smoker | 1,258 (30.2) | 1,065 (10.7) | <0.001 |
| Past smoker | 1,079 (25.9) | 1,543 (15.4) |  |
| Never smoked | 1,768 (42.5) | 7,153 (71.6) |  |
| Missing value | 56 (1.3) | 236 (2.4) |  |
| *Alcohol consumption* | | | |
| Almost daily | 911 (21.9) | 871 (8.7) | <0.001 |
| Occasionally | 2,084 (50.1) | 4,997 (50.0) |  |
| Does not drink | 1,110 (26.7) | 3,893 (38.9) |  |
| Missing value | 56 (1.3) | 236 (2.4) |  |

*t-tests for continuous variables and Fisher’s exact test for categorical variables were performed at a significance level of 0.05.

All values are presented as n (%).

**Supplementary Table 2 Standard mean difference for covariates between the male and female groups before and after adjustment using IPTW**

|  | **Standard mean difference** | |
| --- | --- | --- |
| **Covariates** | **Before IPTW adjustment** | **After IPTW adjustment** |
| Age | 0.24 | 0.24 |
| Age at migraine onset | 0.26 | 0.14 |
| Smoking status | 0.50 | 0.03 |
| Alcoholic intake | 0.28 | 0.08 |
| Obesity | 0.22 | 0.02 |
| Use of acute medications | 0.04 | 0.02 |
| Use of OTC medications | 0.04 | 0.05 |
| Use of preventive medications | 0.15 | 0.01 |
| Work (full-time) | 0.90 | 0.10 |
| Work (part-time) | 0.58 | 0.03 |
| MMD | 0.02 | 0.00 |

IPTW, inverse probability of treatment weighting; MMD, monthly migraine days; OTC, over-the-counter.
